# Quercetin Alleviates Lipopolysaccharide-Induced Cell Oxidative Stress and Inflammatory Responses via Regulation of the TLR4-NF-κB Signaling Pathway in Bovine Rumen Epithelial Cells

**DOI:** 10.3390/toxins15080512

**Published:** 2023-08-21

**Authors:** Maocheng Jiang, Kexin Wang, Yinghao Huang, Xuelei Zhang, Tianyu Yang, Kang Zhan, Guoqi Zhao

**Affiliations:** 1 Institute of Animal Culture Collection and Application, College of Animal Science and Technology, Yangzhou University, Yangzhou 225009, China; 2 Institutes of Agricultural Science and Technology Development, Yangzhou University, Yangzhou 225009, China; 3 Joint International Research Laboratory of Agriculture and Agri-Product Safety, Ministry of Education of China, Yangzhou University, Yangzhou 225009, China

**Keywords:** bovine rumen epithelial cells, inflammation, quercetin

## Abstract

Subacute rumen acidosis (SARA) will cause an increase in endotoxin, which will have a negative effect on the bovine rumen epithelial cells (BREC). Flavonoids are effective in treating inflammation caused by endotoxin. Quercetin is a vital flavonoid widely occurring in fruits and vegetables and has received significant interest as a prospective anti-inflammatory antioxidant. Nonetheless, quercetin’s protective machinery against such damage to BREC induced by lipopolysaccharide (LPS) remains unclear. A combined quercetin and LPS-induced BREC inflammation model was utilized to elucidate the effect of quercetin protecting BREC from LPS-induced injury. After treating BREC with different doses of LPS (1, 5, and 10 μg/mL) for 6 h or 24 h, the mRNA expression of inflammatory factors was detected. Our experimental results show the establishment of the BREC inflammation model via mRNA high expression of pro-inflammatory cytokines in BREC following 6 h treatment with 1 µg/mL LPS. The promotive effect of 80 μg/mL quercetin on BREC growth via the cell counting kit-8 (CCK8) assay was observed. The expression of pro-inflammatory cytokines and chemokines, notably tumor necrosis factor α (TNF-α), Interleukin 1β (IL-1β), IL-6, CC-motif chemokine ligand 2 (CCL2), CCL20, CCL28, and CXC motif chemokine 9 (CXCL9), etc., was significantly reduced by quercetin supplementation. We also analyzed the mRNA detection of related pathways by qRT-PCR. Our validation studies demonstrated that quercetin markedly curbed the mRNA expression of the toll-like receptor 4 (TLR4) and myeloid differentiation primary response protein (MyD88) and the nuclear factor-κB (NF-κB) in LPS-treated BREC. In addition, western blot result outcomes confirmed, as expected, that LPS significantly activated phosphorylation of p44/42 extracellular regulated protein kinases (ERK1/2) and NF-κB. Unexpectedly, this effect was reversed by adding quercetin. To complement western blot results, we assessed p-ERK1/2 and p-p65 protein expression using immunofluorescence, which gave consistent results. Therefore, quercetin’s capacity to bar the TLR4-mediated NF-κB and MAPK signaling pathways may be the cause of its anti-inflammatory effects on LPS-induced inflammatory reactions in BREC. According to these results, quercetin may be utilized as an anti-inflammatory medication to alleviate inflammation brought on by high-grain feed, and it also lays out a conceptual foundation regarding the development and utilization of quercetin in the later stage.

## 1. Introduction

Subacute rumen acidosis (SARA) is a metabolic ailment that easily occurs in high-yield cows. The disturbance of gastrointestinal microflora initiated via a high-concentrate diet is linked with a swift drop in rumen pH and causes subacute rumen acidosis [1]. When the rumen experiences an acidic environment for a long time, gram-negative bacteria dissolve and rupture, releasing LPS [1]. As an inflammatory trigger, LPS binds to the TLR4 receptor, activates inflammation-related signal pathways, and releases many inflammatory factors, such as viz TNF-α, IL-6, and IL-1β etc. The synthesis and release of IL-6 occasionally causes inflammation in related tissues [2,3]. According to research reports by relevant organizations, the occurrence of SARA among dairy cattle is from 11% in early lactation to 26% in the middle of lactation [4,5], and the incidence rate of SARA in some areas is probably higher. SARA can affect dairy cows’ feed intake, production performance, and rumen digestion function, and can cause complications such as diarrhea, hoof leaf inflammation, and tumor gastritis [6,7]. In summary, this common metabolic disease inflicts enormous losses on dairy animal production. Therefore, exploring natural products of anti-inflammatory activity to improve or cure inflammation has become a current research and development hotspot.

Lately, more focus has been placed steadily on the utilization of flavonoids in the feed plants of livestock [8]. There are many reports on the use of different flavonoids in the feeding of animals [9,10]. Previous animal investigations have validated the beneficial impacts of flavonoids on exerting anti-inflammatory as well as anti-oxidative effects in vitro and affects glucose and lipid metabolism [11,12,13,14]. Quercetin, commonly occurring in the human diet, is a component of various flavonoids, such as hesperidium, rutin, and naringenin [15,16]. Some of the common foods that contain quercetin include berries, apples, red onions, and more. Quercetin and its methylated (isorhamnetin, tamarixetin), dehydroxylated (kaempferol), and hydroxylated (rutin) derivatives were found in plasma primarily in conjugated forms, according to a study by Berger [9]. Numerous studies show that quercetin exhibits anti-inflammatory properties [17,18]. Earlier studies have shown that quercetin can effectively impede the mRNA expression of pro-inflammatory factors in human gingival fibroblasts treated with LPS [19]. In other research reports, quercetin can obtain robust anti-oxidation by scavenging free radicals and inhibiting cell proliferation and angiogenesis [20]. Bacterial metabolites and food toxins in the gastrointestinal tract cause damage to the gastrointestinal epithelium. Previous reports proved that the integrity of the gastrointestinal tract is improved by effective components in flavonoids (polyphenols) through various repair mechanisms [21,22]. However, whether quercetin can protect Bovine rumen epithelial cells (BREC) from LPS-induced inflammatory damage is unclear.

In short, quercetin may be a potential therapeutic strategy to alleviate SARA. Therefore, this study investigated the inhibitory effect of quercetin on LPS-induced inflammation of BREC. This lays the foundational knowledge for further exploration and refinement of quercetin-centered functional feed.

## 2. Results

### 2.1. Experiment 1: Effects of Different Concentrations of LPS on BREC

**Figure 1:**
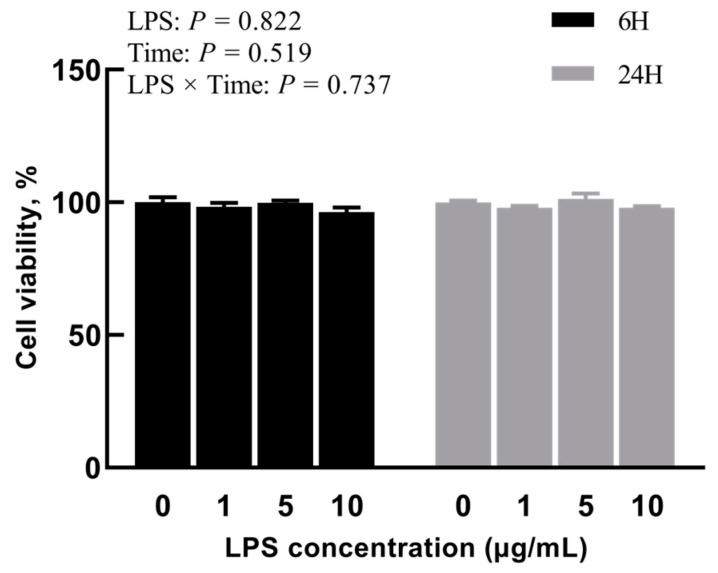
Effects of different concentrations of LPS on BREC viability; bovine rumen epithelial cells were exposed to 1, 5, or 10 µg/mL LPS for 6 h or 24 h; cell viability was assessed by means of the Cell Counting Kit-8 assay (Vazyme, Nanjing, China).

**Figure 2:**
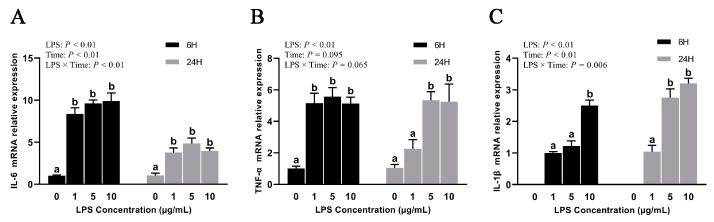
Effects of LPS-stimulating BREC on the expression of inflammatory factor mRNA; (**A**) IL-6 mRNA expression; (**B**) TNF-αmRNA expression; (**C**) IL-1β mRNA expression. The provided data are mean SEM (*n* = 3). Means with different letters (a-b) differed significantly according to treatment group.

To evaluate the cytotoxicity of LPS on BREC after different concentrations of LPS treatment, BREC viability was analyzed via CCK-8 cell viability assay. It was noted that the addition of different concentrations of LPS had a non-significant outcome on the cell activity of BREC in the cases of 6 h and 24 h incubation (*p* = 0.737, Figure 1). Beyond that, the expression of inflammatory factor mRNA by LPS-stimulated BREC from the qRT-PCR point of view was evident. This analysis showed an LPS × Time interaction for IL-6 and IL-1β, with all LPS-treated groups displaying significantly higher IL-6 and IL-1β expression than the control group (*p* < 0.01, Figure 2A,C). The mRNA expression of TNF-α was also considerably higher than the control group (*p* < 0.01), as shown in Figure 2B. Nevertheless, an insignificantly interactive relationship existed between LPS and time concerning TNF-α (*p* = 0.065).

### 2.2. Experiment 2: Effect of Quercetin on LPS-Induced BREC Inflammatory Response

#### 2.2.1. Role of Quercetin in the Cell Viability of BREC

**Figure 3:**
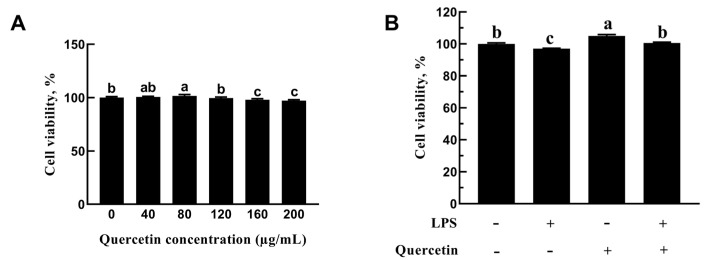
Effects of quercetin on BREC viability. (**A**) Bovine rumen epithelial cells (BREC) were treated with quercetin at various concentrations (40, 80, 120, 160, or 200 µg/mL) for 24 h; (**B**) the Cell Counting Kit-8 assay (Vazyme, Nanjing, China) was used to ascertain cell viability. Means with different letters (a–c) differed significantly according to treatment group.

As evidenced in Figure 3, there was a non-significant change in cell viability which was apparent with quercetin treatment at 40 and 120 µg/mL dosages, whilst promotion of cell viability was noticed at 80 µg/mL dosage (*p* = 0.013, Figure 3). High concentrations of quercetin (160 and 200 µg/mL) significantly inhibit the cell viability of BREC. Thus, quercetin at 80 µg/mL concentration was used for the subsequent studies. To further investigate quercetin’s protective effect on LPS-induced BREC, cells were exposed to quercetin (80 μg/mL) and LPS (1 μg/mL). As seen in Figure 3B, the inhibitory impact of LPS on cell viability was lessened by quercetin treatment (80 μg/mL).

#### 2.2.2. Effect of Quercetin on Oxidative Characteristics of BREC after LPS Stimulation

**Figure 4:**
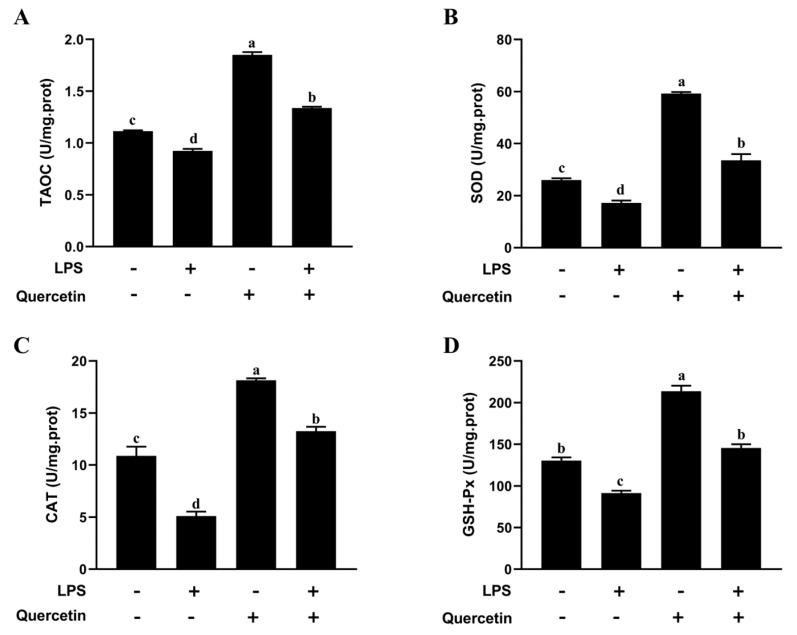
Quercetin efficiently decreased LPS-induced oxidative stress in BREC. BREC was treated with quercetin (80 µg/mL) and 1 µg/mL LPS in 6-well plates for 6 h (1 × 10^6^ cells/mL). TAOC, SOD, CAT, and GSH-Px content levels were detected via kit (**A**–**D**). Means with different letters (a–d) differed significantly according to treatment group.

This section probed quercetin’s capacity to protect BREC from oxidative stress initiated by LPS. Compared with the control group, the group treated with LPS had lower levels of TAOC, SOD, CAT, and GSH-Px. (*p* < 0.05, Figure 4). The outcomes signified that quercetin hampered the LPS-induced oxidation of BREC. Most significantly, compared to LPS alone, quercetin increased (*p* < 0.05) the TAOC, SOD, CAT, and GSH-Px levels in BREC activated by LPS. The outcomes signified that quercetin hampered the LPS-induced oxidation of BREC.

#### 2.2.3. Effect of Quercetin on Pro-Inflammation Factors of BREC after LPS Stimulation

**Figure 5:**
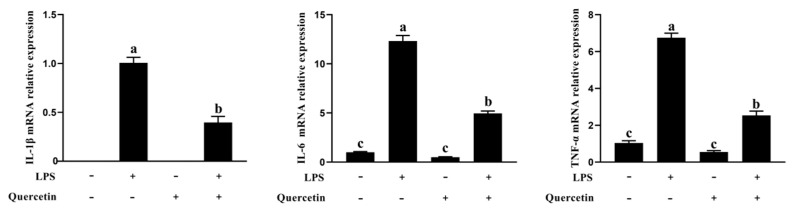
Quercetin efficiently decreased LPS-induced pro-inflammation in BREC. BREC was treated with quercetin (80 µg/mL) and 1 µg/mL LPS in 6-well plates for 6 h (1 × 10^6^ cells/mL). The mRNA levels of TNF-α, IL-1β, and IL-6 genes in the LPS-induced inflammation in the rumen epithelial cells were assessed via qRT-PCR. Means with different letters (a–c) differed significantly according to treatment group.

To investigate the inhibitory effect of quercetin on mRNA expression of pro-inflammation cytokine, the levels were evaluated via qRT-PCR. According to the results in Figure 5, compared with the ctrl group, the mRNA expression of inflammatory factors in the LPS group was significantly increased (*p* < 0.05, Figure 5). This experiment confirmed that quercetin could bar the expression of inflammatory factors induced by LPS (*p* < 0.05). The results demonstrated that quercetin prevented BREC pro-inflammation brought on by LPS.

#### 2.2.4. Effect of Quercetin on the Immune Response of BREC after LPS Stimulation

**Figure 6:**
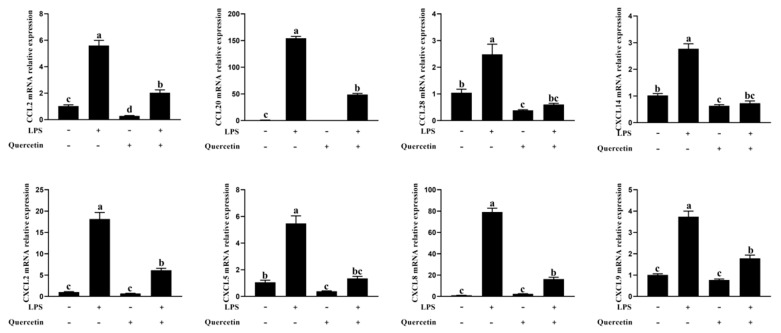
Quercetin efficiently decreased LPS-induced immune response in BREC. BREC was treated with quercetin (80 µg/mL) and 1 µg/mL LPS in 6-well plates for 6 h (1 × 10^6^ cells/mL). The mRNA levels of CCL2, CCL20, CCL28, CXCL14, CXCL2, CXCL5, CXCL8, and CXCL9 genes in the LPS-induced inflammation in the rumen epithelial cells were assessed via qRT-PCR. Means with different letters (a–d) differed significantly according to treatment group.

When compared to unstimulated cells, the BREC with LPS treatment dramatically increased the mRNA expression of the genes for CCL2, CCL20, CCL28, CXCL14, CXCL2, CXCL5, CXCL8, and CXCL9 (*p* < 0.01, Figure 6). Contrarily, in the treatment group that received 80 μg/mL of quercetin while being exposed to 1 μg/mL LPS, the expression of genes linked to immune responses, including CCL2, CCL20, CCL28, CXCL14, CXCL2, CXCL5, CXCL8, and CXCL9, was significantly reduced (Figure 6; *p* < 0.05). According to these outcomes, quercetin decreased BREC’s immune responses induced by LPS.

#### 2.2.5. Effect of Quercetin on TLR4 Signaling Pathway of BREC after LPS Stimulation

**Figure 7:**
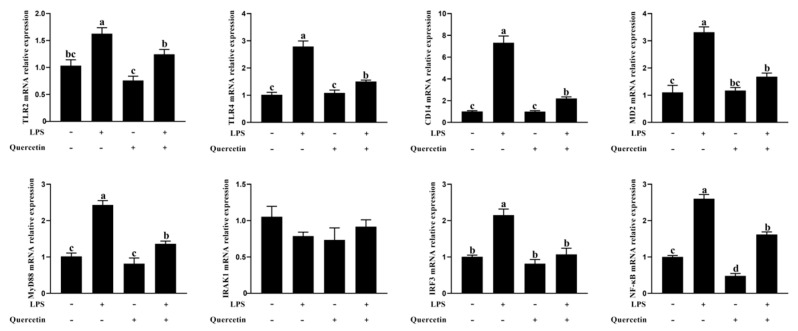
Quercetin efficiently protective BREC was related to the TLR4 signaling pathway. BREC was treated with quercetin (80 µg/mL) and 1 µg/mL LPS in 6-well plates for 6 h (1 × 10^6^ cells/mL). The mRNA levels of TLR4, CD14, MD2, MYD88, IRF3, TLR2, IRAK1, and NF-κB genes in the LPS-treated inflammation in the rumen epithelial cells were assessed via qRT-PCR. Means with different letters (a–d) differed significantly according to treatment group.

Using qRT-PCR, the expression levels of TLR4, TLR2, CD14, MYD88, IRAK1, IRF3, and NF-κB mRNA were measured to determine if quercetin’s protective effect was linked to the TLR4 signaling pathway. TLR4, CD14, MD2, MYD88, IRF3, and NF-κB mRNA levels increased significantly following LPS treatment but decreased significantly in the treatment group receiving 80 μg/mL quercetin amidst 1 μg/mL LPS (Figure 7; *p* < 0.01). According to these data, quercetin’s protective effect was closely linked to the TLR4 signaling pathway.

#### 2.2.6. Effect of Quercetin on ERK1/2 and NF-κB Signaling Pathways of BREC after LPS Stimulation

**Figure 8:**
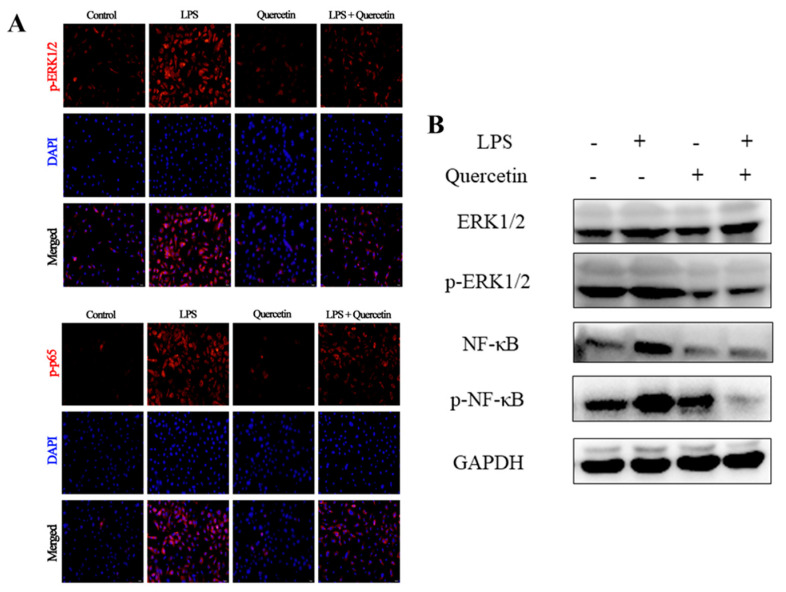
Effect of quercetin on NF-κB signaling pathways in LPS-induced BREC. (**A**) The immunofluorescence for p-p65 and p-ERK1/2 (red) were accomplished, and the nuclear (DAPI; blue) was utilized. Scale bar = 50 µm. (**B**) Western blot analysis of ERK1/2, p-ERK1/2, NF-κB p65, and p-NF-κB p-p65 (*n* = 3).

The protective effect of quercetin on LPS-induced BREC injury was investigated by western blotting and immunofluorescence to ascertain whether it was related to the ERK1/2 and NF-κB signaling pathways. The phosphorylated ERK1/2 and NF-κB levels, essential in an inflammatory response, were higher in LPS-treated BREC than in control groups. Interestingly, the quercetin treatment completely reversed this trend (Figure 8A). These result outcomes match those of western blotting (Figure 8B).

## 3. Discussion

When grain is incorporated in greater quantities in ruminant diets, it produces large quantities of short-chain fatty acids (SCFA) [23]. SARA occurs because of increased SCFA and a drop in the ruminal pH, which causes severe damage to the rumen epithelium [24]. Gram-negative bacteria are lysed more rapidly at low ruminal pH, which raises LPS concentration in the rumen [25]. The excessive production of these LPS can cause inflammation [26]. It has been previously reported that cows with SARA develop inflammation partly due to LPS [27]. Therefore, an in vitro model of BREC was employed to illustrate the direct impacts of LPS on BREC.

For Experiment 1, authors probed the effects of diverse concentrations/dosage of LPS and stimulation time on the cell viability and pro-inflammatory factors of BREC. These results show that the treatment with the addition of diverse dosages of LPS for 6 h or 24 h has no significant effect on cell viability. Thus, our interpretation of this result is that stimulus concentrations lower than 10 µg/mL with LPS do not have a cytotoxic effect on BREC. Our findings are in line with those that have been previously published [28]. During ruminal acidosis, the interaction of BREC with LPS may cause inflammation of the ruminal epithelium. We further investigated the effect of diverse dosages of LPS on the secretion of pro-inflammatory cytokines in BREC. The researchers noted a noticeable increase in mRNA expression of pro-inflammatory cytokines in BREC following 6 h or 24 h of treatment with LPS, in comparison to the control group that did not receive LPS treatment. Therefore, after treatment with LPS (1 µg/mL) for 6 h, the mRNAs of pro-inflammatory cytokines in BREC were highly expressed, and a BREC inflammation model was constructed.

Unlike monogastric animals, the rumen of dairy cows is inhabited by a variety of microorganisms, including rumen protozoa, rumen bacteria, and anaerobic fungi, as well as a few bacteriophages [29]. The ability of quercetin to effectively enter the peripheral circulation of dairy cows is a prerequisite for its ability to function in dairy cows as it does in monogastric animals. A kinetic test of quercetin addition through a rumen fistula showed that the absolute bioavailability of total flavonols (the total of quercetin’s bound and unbound forms, as well as its bound and unbound derivatives) was only 0.1% [29]. It has been shown that quercetin is rapidly degraded to 3, 4-dihydroxyphenylacetic acid and 4-methyl-o-phenol by rumen microorganisms, which may be the reason for the low bioavailability of quercetin [10]. In contrast, the bioavailability of quercetin added to the duodenum of dairy cows was close to that of pigs and other monogastric animals under oral administration [29]. Quercetin has an inhibitory effect on Streptococcus mutant, Streptococcus haematobium, Lactobacillus acidophilus, and Streptococcus teleost, suggesting that it may modulate rumen fermentation by altering the rumen microflora [30]. In ruminants, the rumen holds utmost significance as the primary location for feed breakdown, metabolism, and nutrient uptake. Rumen microorganisms ferment carbohydrates in the diet in the rumen-yielding volatile fatty acids (VFAs), which can provide 60% to 80% of the host’s energy [31]. In vitro simulations of rumen fermentation showed that rumen fermentation parameters were inconsistent with the addition of different doses of quercetin. The addition of 500 mg/L quercetin significantly increased the total volatile fatty acid (TVFA) concentration, and the authors concluded that quercetin stimulated the rumen fermentation [31]. In addition, compared with in vitro fermentation, the rumen-added quercetin was quickly absorbed into the blood or degraded. However, quercetin fermented in vitro could not be discharged into the intestine [10]. Therefore, the significant increase in TVFA concentration by the addition of quercetin in some studies may be caused by the degradation and utilization of quercetin by rumen microorganisms [32]. Research on quercetin in dairy cattle production is still in the preliminary stage, but since quercetin can be degraded by rumen microorganisms and the number of test animals in the above experiments is small, it is necessary to verify the effect of quercetin on dairy cow rumen epithelial cells in vitro, and it lays down a conceptual foundation for the development and utilization of quercetin in the later stage.

Quercetin has been reported to possess antioxidant and anti-inflammatory characteristics [18,33]. Additionally, the imbalance in cell redox due to elevated levels of SCFA might culminate in oxidative stress [34]. Excessive oxidative stress stimulates inflammatory cells, which leads to inflammation. The currently available research points to a connection between oxidative stress and inflammation [35,36]. Inflammatory cells contribute to oxidative stress via reactive oxygen species (ROS), but ROS might additionally promote the generation of inflammatory cytokines [37]. Currently, the regulatory mechanism of quercetin on anti-inflammatory and antioxidant properties is still unclear in BREC. We examined quercetin’s in vitro anti-inflammatory, antioxidant, and mechanistic actions in the present investigation on LPS-stimulated BREC. 

LPS and TLR4 together have been found to activate inflammatory pathways (NF-κB) and cause the generation of ROS, which controls the expression and activity of antioxidant enzymes [38]. In this research, we focused more closely on the antioxidant indicators of BREC. In contrast to the control group, the LPS had significantly lower antioxidant indicators levels in BREC. As anticipated, LPS treatment decreased several indicators of antioxidant enzymes, which is consistent with the current work [39]. Surprisingly, the treatment of 80 μg/mL quercetin at 6 h resulted in an increase of 94, 159, 59, and 44% for antioxidant indicators contents, respectively, compared with the LPS treatment group. Therefore, we speculated that quercetin enhanced antioxidant indicators contents in BREC and that SOD and CAT combined with GSH-Px to scavenge ROS, eventually delaying the senescence of BREC.

We focused on quercetin’s impact on LPS-treated BREC to investigate its anti-inflammatory activities. A pattern-recognition receptor for LPS is TLR4 [40]. TLR4 recognizes LPS and activates the NF-κB signaling pathway to promote the secretion of TNF-α, IL-6, IL-1β, and other inflammatory substances. The accumulation of P-p65 protein in the nucleus has the ability to produce similar substances [41]. Results have been obtained by other researchers who found that TLR4 activates MyD88, NF-κB, and MAPK signaling pathways, and increases production of pro-inflammatory cytokines. [42]. By means of the generation of chemokines and adhesion molecules in response to pathogen invasion, BREC activates the immune response [43]. Chemokines’ main job is to draw a lot of immune cells to the location where they are localized in the tissue. Once there, these cells generated a variety of active constituents that influence tissue inflammation and immunological defects. Our results showed that the levels of chemokines in the LPS group were significantly higher than those in the control group. This indicates that LPS has the ability to enhance the expression of chemokines, which in turn can attract immune cells to process LPS. Compared to the LPS group, chemokine expression dramatically reduced in the quercetin groups. In addition, treatment with quercetin reversed the trend of LPS-induced mRNA expression of pro-inflammatory factors in BREC, indicating quercetin’s ability to subdue LPS-induced inflammation in BREC. Immunofluorescence revealed that quercetin treatment inhibited p-p65′s nuclear entry, while LPS stimulation considerably enhanced p-p65′s nuclear access in BREC. It demonstrated quercetin’s ability to suppress BREC cells’ inflammatory responses by barring the TLR4-NF-κB signaling pathway. Therefore, our study outcomes suggest that the rumen epithelium LPS-induced inflammatory response can be inhibited at 80 µg/mL of quercetin. Additionally, these findings imply that quercetin might be favorable in treating SARA.

## 4. Conclusions

This research has demonstrated that quercetin reduces BREC’s oxidative stress caused by LPS in vitro. Through the interference of the NF-κB signaling pathway, quercetin can lessen the inflammatory response. In summary, by means of the TLR4-NF-κB signaling pathways, treatment with 80 µg/mL quercetin can protect BREC’s normal physiological function from LPS-induced oxidative stress and inflammatory response. This research will establish a theoretical foundation for investigating the pharmacodynamic mechanisms of quercetin and its potential applications in the deterrence and management of SARA in dairy cows.

## 5. Materials and Methods

### 5.1. Cell Culture

BREC was procured from Yangzhou University’s Institute of Animal Culture Collection and Application in China, and the development of immortalized BREC was based on findings from an earlier investigation [44]. By using lentivirus transfection, authors were able to create immortalized BREC from the original. Furthermore, authors recognized immortalized BREC via western blot and qRT-PCR. BREC were cultured in DMEM/F12 medium. The medium was supplemented with 10% fetal bovine serum (Gibco, Grand Island, NY, USA), 100 U/mL penicillin, and 100 µg/mL streptomycin (Shenggong Biotechnology, Shanghai, China). The culture environment was 37 °C and 5% carbon dioxide (CO_2_).

### 5.2. Experiment 1: Establishment of an LPS-Induced BREC Inflammatory Model

#### 5.2.1. Effects of Different Dosage/Concentrations of LPS on BREC

According to Wang’s description [44], the impact of LPS on BREC toxicity was investigated. First, 96-well plates were used to seed the BREC (5 × 10^3^ cells/well). Post 12 h cells were incubated for 6 h or 12 h with 0, 1, 5, or 10 μg/mL LPS (n = 5). The cells were subsequently exposed to 10 L of CCK-8 reagent, and they were incubated for a further three hours at 37 °C with 5% CO_2_. The enzyme labeling instrument (Thermo Fisher, MultiskanFC, Shanghai, China) was used to quantify the OD values at 450 nm.

#### 5.2.2. Effects of LPS on mRNA Levels of Inflammatory Factors in BREC

The BREC was cultivated at 37 °C with 5% CO_2_ after being sown in 6-well plates with 1 × 10^6^ cells per well. Three experimental groups were produced using DMEM/F12 medium: (1) Control; (2) DMEM/F12 medium + LPS (1, 5, and 10 µg/mL) for 6 h; and (3) DMEM/F12 medium + LPS (1, 5, and 10 µg/mL) during 24 h collection of BREC for pro-inflammatory factors (IL-1β, IL-6, and TNF-α) mRNA expression.

### 5.3. Experiment 2: Effect of Quercetin on LPS-Induced BREC Inflammatory Response

#### 5.3.1. Proliferative Activity Analysis

The impact of various quercetin concentrations on the proliferative activity and toxicity analyses of BREC was identified using the CCK-8 kit. The procedure was the same as that used in Experiment 1. BREC were added and adjusted to 1 × 10^5^ cells/mL on 96-well culture plates. The media was taken out of the culture after 12 h. Quercetin (0, 40, 80, 120, 160, or 200 µg/mL, 100 µL/well; Sigma-Alrich, St. Louis, MO, USA) was used to stimulate the BREC for 24 h. CCK8 was then added to each experimental group (µL/well; Vazyme, Nanjing, China) and cultured for 3 h at 37 °C with 5% CO_2_. Utilizing a microplate reader (Thermo Fisher, MultiskanFC, Shanghai, China), the OD values were measured at 450 nm.

#### 5.3.2. Experimental Design and Treatment

BREC were added and adjusted to 1 × 10^6^ cells/mL on 6-well culture plates. The BREC were separated into the following 4 groups, namely (1) DMEM/F12 medium for the control group; (2) 1 µg/mL LPS for LPS group; (3) 80 µg/mL quercetin for Q group; and (4) 1 µg/mL LPS + 80 µg/mL quercetin for LPS + Q group), cultured for 24 h, and then a fresh medium was used. The cells from each group were harvested independently after 6 h of incubation.

#### 5.3.3. RNA Isolation and cDNA Synthesis

This was completed per the operation instructions of the RNA extraction kit (Takara, code: RR036A, Dalian, China). CDNA was obtained using a reverse transcription kit (Takara, Tokyo, Japan). SYBR ^®^ Premix Ex Taq ™ II kit (Takara, Dalian, China) was used for real-time PCR detection. β-actin and GAPDH are already known to be suitable for BREC and were estimated by the 2^−ΔΔCT^ method [28]. Similar result outcomes were obtained and only the results using GAPDH as a normalizer were shown. All primers in the experiment are itemized in Table 1.

#### 5.3.4. Antioxidant Analysis

After the digestion of BREC, cell suspension density was to be adjusted to 1 × 10^6^ cells/mL. The cell suspension was inoculated into a 6-well plate. When cells proliferated to approximately 80–90%, LPS and quercetin were added. The cells were collected after 6 h. According to the manufacturer’s instructions, we used the antioxidant related Assay Kit (JianCheng Bioengineering Institute, Nanjing, China) to measure the levels of TAOC, MDA, GSH-PX, SOD, and CAT in cell samples.

#### 5.3.5. Immunocy to Fluorescence

BREC was added to an 8-well chamber slide (Thermo Scientific, Lab-Tek^TM^II, Code No.154534, New York, NY, USA) and adjusted to 2 × 10^3^ cells/mL. Cell proliferation was approximately 80–90% and treated with 1 µg/mL LPS and 80 µg/mL quercetin. Next, the slides were washed thrice using PBS for 3 min each time, fixed at room temperature for 30 min with 4% paraformaldehyde, rinsed thrice with PBS, and then re-rinsed with ethylenediaminetetracetic acid disodium (EDTA-Na_2_; 95 °C, 5 min) for antigen retrieval. PBS containing 3% horse serum was introduced drop by drop onto the slides and blocked at room temperature for 1 h. The blocking solution was drawn out, followed by primary antibody addition and incubation all night at 4 °C. The selection of the first antibody refers to Wang’s experiment [44]. Next, it was the addition of anti- rabbit immunoglobulin G (IgG) in combination with fluorescein isothiocyanate (Cy3) (1:2000; Abcam, Shanghai, China) and it was cultured at room temperature for 1 h to prevent light exposure. Then, it was washed thrice with PBS before DAPI staining. After 8 min, the cells were cleaned thrice with PBS and visually appraised via a confocal laser scanning microscope (Olympus, Tokyo, Japan).

#### 5.3.6. Western Blotting

The BCA kit (Beyotime, Beijing, China) was utilized to compute protein concentrations in cell samples for total protein extraction. Protein samples were prepared according to Wang’s method [44]. Following blocking with 5% horse serum, the membranes were incubated with the primary antibody and 5% horse serum in Tris-buffered saline containing Tween (TBS-T: 10 mM Tris–HCl, pH 7.5, 150 mM NaCl, 0.05% Tween 20) and kept all night at 4 °C. The ensuing primary antibodies were acquired from phosphorylation-ERK1/2 (p-p44/42) rabbit mAb (1:2000; Cell Signaling Technology, Shanghai, China), ERK1/2 (p44/42) rabbit mAb (1:1000; Cell Signaling Technology, Shanghai, China), NF-κB (p65) rabbit mAb (1:1000; Cell Signaling Technology, Shanghai, China), phosphorylation-NF-κB (p-p65) rabbit mAb (1:1000; Cell Signaling Technology, Shanghai, China), and GAPDH (1:1000; Cell Signaling Technology, Shanghai, China). Goat anti-rabbit IgG (1:5000; CST) were the secondary antibody used, and it was conjugated with horseradish peroxidase (HRP). The target bands were spotted employing pierce ECL Plus western blotting substrate or super signal west femto maximum sensitivity substrate from Thermo Scientific.

### 5.4. Statistical Analysis

The data outcomes were presented as means ± standard error (SEM). For scenarios involving two or more variables, group mean comparisons were conducted using two-way ANOVA, followed by either Tukey’s or Sidak’s multiple comparison tests. The obtained experimental data were examined utilizing the SPSS Statistics program. Values with the same letter do not show any differences that are significant (*p* > 0.05), while values with different letter values show differences that are significant (*p* < 0.05).

## Figures and Tables

**Table 1:** Primers for real-time PCR analyses.

Gene	Primer Sequence, 5′ to 3′	Accession Number	Size (bp)
GAPDH	F: GGGTCATCATCTCTGCACCT R: GGTCATAAGTCCCTCCACGA	NM_001034034.2	176
IL-1β	F: CAGTGCCTACGCACATGTCT R: AGAGGAGGTGGAGAGCCTTC	NM_174093.1	209
IL-6	F: CACCCCAGGCAGACTACTTC R: TCCTTGCTGCTTTCACACTC	NM_173923.2	129
TNF-α	F: GCCCTCTGGTTCAGACACTC R: AGATGAGGTAAAGCCCGTCA	NM_173966.3	192
CXCL5	F: TGAGACTGCTATCCAGCCG R: AGATCACTGACCGTTTTGGG	NM_174300.2	193
CCL2	F: GCTCGCTCAGCCAGATGCAA R: GGACACTTGCTGCTGGTGACTC	NM_174006	171
CXCL2	F: CCCGTGGTCAACGAACTGCGCTGC R: CTAGTTTAGCATCTTATCGATGATT	NM_174299.3	204
CXCL8	F: TGGGCCACACTGTGAAAAT R: TCATGGATCTTGCTTCTCAGC	NM_173925.2	136
CXCL9	F: ACTGGAGTTCAAGGAGTTCCAGCA R: TCTCACAAGAAGGGCTTGGAGCAA	NM_001113172.1	127
CCL20	F: TTCGACTGCTGTCTCCGATA R: GCACAACTTGTTTCACCCACT	NM_174263.2	172
CCL28	F: GCTTCTGGAAAGAGTGACAACGT R: AGGATGACAGCAGCCAAGTC	NM_001101163.1	72
CXCL14	F: AATGGTACAACGCCTGGAAC R: GTTCCAGGCGTTGTACCATT	NM_001034410.2	153
TLR2	F: CAGGCTTCTTCTCTGTCTTGT R: CTGTTGCCGACATAGGTGATA	NM_174197.2	140
TLR4	F: GACCCTTGCGTACAGGTTGT R: GGTCCAGCATCTTGGTTGAT	NM_174198.6	103
CD14	F: CAGTATGCTGACACAATCAA R: AGTTCCTTGAGACGAGAGTA	NM_174008.1	122
MD2	F: GGAGAATCGTTGGGTCTGC R: GCTCAGAACGTATTGAAACAGGA	NM_001046517.1	92
MyD88	F: TCATTGAGAAGAGGTGCCGT R: TGGCTTGTACTTGATGGGGAT	NM_001014382.2	146
IRF3	F: TTGTGAACTCAGGAGTCAGG R: TGGGCTCAAGTCCATGTCAC	NM_001029845.3	125
IRAK1	F: CCTCAGCGACTGGACATCCT R: GGACGTTGGAACTCTTGACATCT	NM_001040555.1	103
NF-κB	F: AACAACCCCTTCCAAGTTCC R: CTCCCAGAGTTCCGATTCAC	NM_001080242	203

F, forward; R, reverse.

## Data Availability

Not applicable.
